# Our Contemporaries

**Published:** 1912-12

**Authors:** 


### OUR CONTEMPORARIES.

---

The *American Practitioner* of October, is devoted almost entirely to articles concerning public health—a most excellent number.

---

The *Detroit Medical Journal* of October, contains a symposium on the question of fee splitting. None of the writers seem aware that the crusade against this evil was initiated by Dr. M. D. Mann of Buffalo, in a paper presented to the Medical Society of the Co. of Erie, Feb. 21, 1910. Dr. J. H. Carstens makes the somewhat novel plea that the surgeon, in abandoning general practice ,gives hundreds of dollars to each of his potential competitiors, thereby virtually paying a commission in advance.

---

The *Old Dominion Journal of Medicine and Surgery* of August, and the *Medical Fortnightly,* of Nov. 11, 1912, abstract Col. Crego's article on Meningitis from our May issue.

---

The State Charities Aid Association has started to publish from its headquarters, 105 E. 22d St., New York, the "*S. C. A. A.*

*News*" (sic), an 8-page bulletin intended for free distribution, containing much interesting information, especially regarding the fight against tuberculosis. We would advise our readers to apply for it.

---

The *Medical Times*, Nov., 1912, contains an interesting symposium on the question of a medical editor for a newspaper. The editor, Dr. Simon Baruch, Medical Editor of the *Sun*, Oswald Garrison Villard, President of the *Post*, James Kelly, General Manager of Chicago *Tribune*, Frederick Roy Martin, Assistant General Manager of the Associated Press, Leigh Reilly, Editor of the Chicago *Evening Post*, Jay B. Benton, City Editor of the Boston *Evening Transcript*. Melville E. Stone, General Manager of the Associated Press, and Talcott Williams, President of the Pulitzer School of Journalism, all contribute and all speak on the affirmative. It is noteworthy that most of the non-medical contributors emphasize the difficulty of finding the right man. The writer, having at one time made some attempt at writing for literary and news periodicals, with an average of about 90% of rejections, can appreciate the fact that it is difficult to suit the popular appetite for reading matter, or at least the standards of those who judge whether it would be suited or not. Those of us who have been unsuccessful can find some comfort in the historic fact that many acknowledged master-pieces have been at first rejected and that well known literary favorites who occasionally try the experiment of sending a story under an unknown name, usually find that it is rejected by the very men who are clamoring for their wares. The writer had one article rejected by several literary magazines. It was then published in a medical journal and published in abstract, with illustrations, by one of the New York papers. We can also derive comfort from the professional rating of physicians who have gone into "writing" for the lay press, as a business. But, however, we try to sooth our vanity, the fact remains that medical men, as a rule, are not satisfactory as writers for the non-medical press. Possibly the reason is indicated by the fact that we call our work "articles" or "reports" and that the newspaper man calls his work "stories." That is to say, we neglect literary style—too often grammar—try to condense, and to exclue every extraneous allusion, however it might please the fancy; in short we concentrate on the intrinsic interest of the case or subject. The magazine and press writer, on the other hand, except in the matter of murders, suicides, personals, etc., deals with occurrences which are not—or which he assumes are not—of interest to his readers, except as he makes them so. In this effort, he very justly feels that accuracy may be sacraficed, whereas the medical man is trained by habit to be a slave to accuracy. Then, too, the latter does not speak the ordinary language of the country but a mixture of this with a technical vocabulary of some 40,000 words. The writer remembers, during the time that he was studying blood vessels, quite unconsciously mentioning a walk to the "bifurcation" of Huron St. (Ann Arbor). The knack of stating technical facts, accurately, in ordinary language, and of explaining a necessary technicality in plain English, without pedantry, is something that few of us possess. The faculty is quite as rare as the ability to use two languages idiomatically.

